# Endogenous mediators in regulating primary dysmenorrhea

**DOI:** 10.3389/fcell.2026.1756355

**Published:** 2026-03-31

**Authors:** Wenwen Duan, Yingtao Ma, Qinjian Dong, Quanwu Wang, Yijie Xie, Guangmei Liu, Chao Wang, Min He

**Affiliations:** 1 Department of Acupuncture and Tuina, Chengdu University of Traditional Chinese Medicine, Chengdu, Sichuan, China; 2 Department of Pediatrics, The Third Affiliated Hospital of Changchun University of Chinese Medicine, Changchun, Jilin, China; 3 Department of Tuina, The Affiliated Hospital of Changchun University of Chinese Medicine, Changchun, Jilin, China; 4 Department of Anus and Intestine Surgery, Maoming Hospital of Guangzhou University of Traditional Chinese Medicine, Maoming, Guangdong, China; 5 Sub-Health Department, Sichuan Integrative Medicine Hospital, Chengdu, Sichuan, China; 6 Northeast Asia Research Institute of Traditional Chinese Medicine, Changchun University of Chinese Medicine, Changchun, Jilin, China

**Keywords:** antioxidants, endocrine hormones, endogenous mediators, immunomodulatory factors, neurotransmitters, primary dysmenorrhea

## Abstract

Primary dysmenorrhea is currently an unresolved public health problem worldwide, which suffers the patients comprehensive symptoms. Currently, more and more studies have been focusing on biomarker exploration for diagnosis and target therapies of the primary dysmenorrhea. In this paper, we integrated and summarized the reported potential endogenous mediators of primary dysmenorrhea, which refer to endocrine mediators such as prostaglandins, vasopressin, estradiol, oxytocin, endothelin-1, etc., that contribute to inflammatory response and regulation of endometrial myometrial, vasoconstriction, as well as the onset of pain. With the help of current techniques, such as metabolomics (for, *e.g.,* serum oxylipins), proteomics, as well as mass spectrometry imaging and brain imaging techniques, there will be great potential in finding more reasonable and promising markers of primary dysmenorrhea, which will greatly improve the early diagnosis and personalized therapies of primary dysmenorrhea in clinics.

## 1 Introduction

Primary dysmenorrhea (PD) is affecting 50%–90% of women, defined as a gynecological disease with painful menstruation in the absence of pelvic pathological lesions in the lower abdomen, such as endometriosis, fibroids, adenomyosis, and congenital anatomic abnormalities (Guimarães and Póvoa, 2020; Yang et al., 2022). Half of the patients describe their pain as moderate to severe, and experience many other clinical symptoms such as nausea, fatigue, vomiting, headache, and even fainting (Guimarães and Póvoa, 2020; Zhang et al., 2022; Pan S. et al., 2022). Long-lasting dysmenorrhea can cause also depression, anxiety and other mood disorders (Valedi et al., 2022). In terms of treatment costs, the heavy financial burden directly affect the patient’s choice of medical treatment and the degree of awareness of the disease (Holmes et al., 2021). Currently, most of the patients regard the primary dysmenorrhea as a normal phenomenon of menstruation and seldom consult actively a medical professional, and do not raise sufficient awareness that it may develop into other severe diseases (Parra-Fernández et al., 2020; Kho and Shields, 2020; Öztürk and Güneri, 2021; Clemenza et al., 2021). In addition, primary dysmenorrhea has also a significant impact on social healthcare planning (Molla et al., 2022). While such a high prevalence disease is still with less understanding on its diagnosis, and the unclear pathogenic markers results in the inadequate awareness and treatments of this disease.

Recently, more and more experimental studies are focusing on the developed methods for detecting the endogenous markers, among of which the primary dysmenorrhea studies are increasingly emerging. This review summarizes the potential endogenous markers of primary dysmenorrhea reported in recent articles, including markers that relate to regulation of inflammation, oxidative stress, hormones, neurotransmitters, etc., so that to provide precise clinical treatment for a better personalized healthcare.

## 2 Endogenous mediators in regulating inflammatory and pain intensity in PD

The functions of the endometrium are related to implantation, angiogenesis, cellular differentiation and tissue repair, all of which can be regulated by various endogenous substances including antioxidants, endocrine hormones (Szmidt et al., 2020; Jabbour et al., 2006), immunomodulatory factors, neuropeptides, etc. These substances regulate the shedding of endometrial tissue from the uterus involved in the occurrence of menstruation and the pathological process of primary dysmenorrhea (Critchley et al., 2020; Ashwell, 2022; Payne et al., 2019). For instance, the hypothalamic-pituitary-gonadal axis stimulates the anterior pituitary to secrete follicle-stimulating hormone (FSH) and luteinizing hormone (LH), thereby promoting follicular growth and maturation (Jaleel et al., 2022). The sudden withdrawal of progesterone in the late luteal phase triggers an inflammatory cascade characterized by increased production of endometrial inflammatory mediators, including cytokines (e.g., IL-1β), chemokines, and matrix metalloproteinases (MMPs), and these processes are mediated by transcription factors such as nuclear factor-kappa B (NF-κB) (Mauracher et al., 2021; Jain et al., 2022; Watters et al., 2021). When the uterus is exposed to these inflammatory mediators, leukocytes are recruited into the endometrium and promote the inflammatory process of menstruation (Critchley et al., 2020; Zhu et al., 2022). Central neurotransmitters are recognized to be involved in a wide range of regulatory activities including neuroprotection, neural-glial interactions, central control of autonomic functions, control of vessel tone and angiogenesis, pain and mechanosensory transduction, as well as the physiological senses, and are thus closely associated with dysmenorrhea. For instance, the peptidergic nerves that are present in the uterus can act on the myometrium and uterine vessels, causing contraction and ischemia (Sjöberg, 1979). Endogenous opioids, serotonin (5-hydroxytryptamine, 5-HT), the endocannabinoid system, as well as altered N-methyl-D-aspartate (NMDA) receptor signaling, dysregulated noradrenaline secretion, and glial factors have been implicated in the pathophysiology of primary dysmenorrhea (Jaleel et al., 2022; De Deurwaerdere and Di Giovanni, 2021).

### 2.1 Hypoxia and oxidative stress

Oxidative stress is considered to be a state of imbalance between the over-production and insufficient elimination of reactive oxygen species (ROS) (Hussain et al., 2016). ROS are produced in living cells and regulate basal metabolic processes in aerobic organisms, thus playing key roles in vascular, cardiac and neurological and reproductive diseases detected in *in serum* (Münzel et al., 2017; Ya et al., 2020; Afza et al., 2023). Excessive ROS can induce inflammation by promoting the activation of transcription factors (e.g., NF-κB) (Vitale et al., 2013). Hypoxia is an inevitable cellular stress in many diseases (Chandimali et al., 2025). Hypoxia is an inevitable cellular stress in many diseases. Dysmenorrhea is reported to be associated with uterine ischemia and hypoxia to induce the ROS, which further disrupt the productions of lipids, nucleic acids, proteins in living cells and causes these substances to peroxidation (Orimadegun et al., 2019). I It has been demonstrated that hypoxia has negative effects on estrogen biosynthesis, pro-inflammatory cytokine production, angiogenesis and immune function, and hypoxia-inducible factor-1α (HIF-1α) expression is significantly increased in endometriosis (Zhan et al., 2016; Li W. N. et al., 2021). Recently, elevated indicators (such as malondialdehyde (MDA), nitric oxide (NO), adrenomedullin (AM) (Dikensoy et al., 2008), asymmetric dimethylarginine (ADMA), Heme oxygenase-1 (HO-1), and 3-nitrotyrosine (3-NT)) have been detected in serum of women with primary dysmenorrhea (Núñez-Troconis et al., 2021). Additionally, elevated high-sensitivity C-reactive protein (hs-CRP) and perturbation of serum prooxidant–antioxidant balance (PAB) were observed in women with primary dysmenorrhea (Orimadegun et al., 2019; Bahrami et al., 2020; Ghayour-Mobarhan et al., 2009).

### 2.2 Prostaglandins (PGs)

The prostagnoids (including endoperoxides, classical prostaglandins, thromboxane and prostacyclin) (Berisha et al., 2024; Zeng et al., 2023) are formed by metabolism of unsaturated fatty acids and are well known as their broad biological potencies such as intense vasomotor activity, effects on gastrointestinal, bronchial and uterine smooth muscle, as well as on the platelet system. Among which, the prostaglandins are considered as important inflammatory mediators that participate in the development of the primary dysmenorrhea (Bernardi et al., 2017; Barcikowska et al., 2020). The presence of these endogenous components can shrink muscle muscles, thus are often considered as menstrual stimulants. They can promote the shedding of the endometrium and the rhythmic contraction of the uterus, while the excessive of which may cause also strong pain caused by spastic contractions of uterine muscles (Pickles, 1963). Among these menstrual stimulants, PGF_2α_ and PGE_2_ are two highly abundant and active PGs present in menstrual fluid (Eglinton et al., 1963). Most patients with dysmenorrhea were reported to be with significantly higher levels of PG in their uterus than those subjects without dysmenorrhea, which may due to the abnormal PG productions by the autocrine and paracrine pathways (Chan and Hill, 1978; Lundström et al., 1979; Chan et al., 1979).

At the end of the luteal phase, decreased progesterone levels lead to lysosomal membrane destabilization, facilitating the release of phospholipases (particularly phospholipase A_2_) and subsequent hydrolysis of membrane phospholipids. These processes result in the liberation of arachidonic acid (AA)—from cellular membranes (Barcikowska et al., 2020; Seo and Oh, 2017; Wang et al., 2021; Dawood et al., 1987; Draper et al., 2018). In the presence of cyclooxygenase (COX), AA is oxidized to form PGG_2_, which is subsequently reduced to PGH_2_. Various prostaglandin synthases then act on PGH_2_ to generate bioactive prostaglandins (Barcikowska et al., 2020; Sales and Jabbour, 2003; Song et al., 2025). PGE_2_ exhibits both uterine contractile and relaxant effects, whereas PGF2α predominantly promotes myometrial contraction (Li W. J. et al., 2021). An increased PGF_2α_/PGE_2_ ratio has been reported during menstruation in women with dysmenorrhea (Pan S. et al., 2022; Mrugacz et al., 2013). Furthermore, an increased ratio of plasma TXB_2_ (a metabolite of the platelet aggregator TXA_2_)/6-keto-PGF1α (a metabolite of vasodilator PGI_2_) in a mouse model of oxytocin-induced primary dysmenorrhea (Yang et al., 2015). These findings suggest that elevated prostaglandin levels not only mediate uterine vasoconstriction but may also serve as biochemical indicators of disease severity (Yang et al., 2015).

### 2.3 Vasopressin (AVP)

Vasopressin, also known as antidiuretic hormone, is a peptide hormone secreted by the posterior pituitary gland. Arginine vasopressin (AVP) is the predominant form of vasopressin in humans (Sparapani et al., 2021; Strömberg et al., 1984). The AVP can act on the reproductive system; it can be produced by the fetus during late gestation and may also be locally synthesized in the uterus, including the decidua of the endometrium (Sparapani et al., 2021; Akerlund, 2002; Arrowsmith, 2020). The plasma level of vasopressin in women with premenstrual pain and dysmenorrhea has been reported to be higher than those in women without dysmenorrhea. Before menstruation begins, the AVP can act on the myometrium and bind to vasopressin V1a receptors which are distributed on uterine smooth muscle cells. Then Gq/11-type G-protein stimulates the activity of phospholipase C, and further hydrolyzes phosphatidylinositol 4,5-bisphosphate (PIP_2_) into inositol 1,4,5-trisphosphate (IP_3_) and diacylglycerol (DAG). This results to excessive increase of intracellular calcium and protein kinase C levels, promoting the uterine contraction and causing uterine ischemia and hypoxia (Strömberg et al., 1984; Akerlund et al., 1979; Demiselle et al., 2020; Yoshimura et al., 2021; Bossmar et al., 1995a; Liccardo et al., 2022). Therefore, AVP as a potential biological indicator may also reflect the primary dysmenorrhea.

### 2.4 Estradiol and progesterone

Estradiol and progesterone are steroid hormones secreted by the ovaries, which influences each other and maintain a dynamic equilibrium relationship (King and Critchley, 2010; Bossmar et al., 1995b). Current studies often explore these two together during primary dysmenorrhea studies. The estrogen at supraphysiological doses has been reported to cause dilation and edema of endometrial glands (Egger and Kindermann, 1974). The estradiol can activate peroxidase and promote the production of prostaglandins, thus may cause uterine contraction (MacLean and Hayashi, 2022; Ylikorkala et al., 1979). The estradiol can also promote over-expression of transient receptor potential vanilloid 6 (TRPV6), which facilitates the large influx of calcium ions into the cell membrane, thus enhances the spontaneous contraction of the myometrium (Andersson, 1988; Uchida and Izumizaki, 2021). The Extracellular Regulated Kinase 1/2 (ERK1/2) -p90 ribosomal S6 kinase (RSK) signaling pathway, as well as the estrogen signaling pathway, can synergistically regulate estrogen homeostasis in the uterus by mediating the estrogen receptor. Excess estrogen occurs also by disruption of the mitogen-activated protein kinase (MAPK) pathway, which has been demonstrated in the breast cancer research (Lannigan, 2022).

A mid-cycle peak in estradiol triggers a peak in luteinizing hormone (LH). The LH stimulates cells in the corpus luteum and convert cholesterol to pregnenolone by cholesterol side-chain lyases in the mitochondria, after which progesterone is further converted from pregnenolone via the enzyme 3β-hydroxysteroid dehydrogenase (Prior, 2020; Sundström-Poromaa et al., 2020). The converted progesterone is then metabolized into different products, thereby inhibiting the production of prostaglandins by controlling the expression of estradiol, and to exert analgesic effects by binding to progesterone receptors, membrane receptors, and GABA receptors (Pan S. et al., 2022; Sundström-Poromaa et al., 2020; Kolatorova et al., 2022). Insufficient progesterone or too much estradiol may both cause excessive curvature of the uterine glands (Good and Moyer, 1968). Moreover, progesterone and estradiol can affect uterine endocrine function by modulating the secretion of Endothelins (EDNs). The Endothelin Receptor Type B (EDNRB) located on vascular smooth muscle are preferentially exerted following progesterone withdrawal (Keator et al., 2011), resulting in the stimulation of uterine smooth muscle contraction, reduction of blood flow, and then causing pain.

### 2.5 Oxytocin (OT)

Oxytocin is a neuropeptide hormone produced by hypothalamic OT neurons (Liu et al., 2022), the synthesis and secretion of which can be regulated by estrogen (Jirikowski et al., 2018). Classically, elevated OT levels were thought to cause dysmenorrhea. In the normal endometrium of nonpregnant women, OT specifically binds to OT receptors in the endometrial glandular epithelium, activating Gαq/11 protein, which further activates phospholipase C-β (PLC-β). Then the PLC-β hydrolyzes phosphatidylinositol 4,5-bisphosphate (PIP_2_) into inositol 1,4,5-trisphosphate (IP_3_) and diacylglycerol (DAG). The IP3 promotes calcium release from the sarcoplasmic reticulum, while the DAG activates protein kinase C (PKC) which in turn activates the mitogen-activated protein kinase (MAPK) cascade (Shojo and Kaneko, 2000; Takemura et al., 1993; Arrowsmith et al., 2014). These lead to myometrial contraction and primary symptoms resembling dysmenorrhea (Liedm et al., 2008).

However, different views have emerged in recent years. A recent clinical study reported that serum OT levels in women with dysmenorrhea were significantly lower than that in women without dysmenorrhea, and the level of OT was negatively correlated with the degree of dysmenorrhea (Liu et al., 2022; Oladosu et al., 2020). This study indicates that dysmenorrhea may result from insufficient OT rather than OT overproduction. Another recent study supports potential analgesic and anti-inflammatory effects of OT by stimulating the vagus nerve and affecting the autonomic nervous system (Iovino et al., 2021). However, most of the current oxytocin studies focus mainly on the reproductive system related to pregnancy. More experimental studies on the relation between the oxytocin and primary dysmenorrhea are needed.

### 2.6 Endothelin-1 (ET-1)

Endothelin-1 (ET-1) is a strong human uterine vasoconstrictor that enhances the contractile activity of the non-pregnant myometrium and regulates menstrual bleeding and promote endometrial repair and proliferation (Word et al., 1990), by directly acting on endothelin receptors of endometrial epithelial cells and blood vessels (Keator et al., 2011; Marsh et al., 1996). The secretion of ET-1 can be increased by the stimulation of E2 during the proliferative phase and by the withdrawal of progesterone during the secretory phase (Bodelsson et al., 1992; Tanfin et al., 2011). ET-1 has been reported to induce vasospasm by acting on the spiral arterioles of the endometrium (Economos et al., 1992), and the ET-1 and PGF2α can stimulate the synthesis and secretion of each other in cattle and sheep, which can synergistically lead to luteolysis and further exacerbate dysmenorrheic symptoms (Milvae, 2000). These findings suggest that ET-1 may serve as a potential endogenous marker for regulating inflammation and pain in primary dysmenorrhea.

### 2.7 Cytokines

Cytokines, as key mediators regulating inflammatory and immune responses, are also mentioned in the review by Barcikowska et al. (2020). Cytokines are secreted by a variety of immune cells including lymphocytes, macrophages, natural killer (NK) cells, mast cells and stromal cells. Cytokines are classified as tumor necrosis factors (TNFs), interleukins (ILs), interferons (IFNs), colony-stimulating factors (CSFs), and transforming growth factors (TGFs). These cytokines can be either pro-inflammatory or anti-inflammatory (Liu et al., 2021; Xing and Wang, 2000). Studies have shown that the occurrence of primary dysmenorrhea is closely related to the immune dysfunction of patients, which is manifested in the imbalance between pro-inflammatory factors and anti-inflammatory factors in the immune regulation network (Ma et al., 2013; Xia et al., 2025). The imbalance of immune function in patients with primary dysmenorrhea may involve macrophage polarization. When macrophages are polarized to the M1 phenotype, they secrete a large number of pro-inflammatory factors (such as TNF-α, IL-1β, IL-6), thereby exacerbating the inflammatory response; when polarized to the M2 phenotype, they mainly secrete anti-inflammatory factors (such as IL-10, TGF-β), which help to reduce inflammation (Park et al., 2025). In a case-control study, women with primary dysmenorrhea were reported to have higher pro-inflammatory TNF-α level and lower anti-inflammatory cytokines on the first day of menstruation than healthy controls (Ma et al., 2013). TNF-α is reported to induce pain and regulate the inflammatory response by promoting luteolysis, stimulating the production and release of prostaglandin, and inducing uterine contractile activity (Ma et al., 2013; Calleja-Agius et al., 2009). An animal experiment study found that IL-1β exhibits a high expression level in the mouse model of primary dysmenorrhea, and Peony Pollen reduced the expression levels of IL-1β (Yang et al., 2023). Another animal study demonstrated significantly elevated levels of IL-1β protein expression in a rat model of primary dysmenorrhea (Xue et al., 2023). IL-1β can trigger pain (Xue et al., 2025). IL-6 is expressed in the endometrium by epithelial and stromal cells, and plays multiple roles in regulating immune system, tissue regeneration, and metabolism, acting on endometrial epithelial and stromal cells (Kang et al., 2019; Millrine et al., 2022; Laird et al., 1993; Laird et al., 1994). Elevated serum IL-6 levels were reported in Taiwanese women with dysmenorrhea compared to those without, indicating the pro-inflammatory role in dysmenorrhea (Yeh et al., 2004).

### 2.8 Chemokines

The chemokines as a class of small cytokines can induce targeted chemotaxis of nearby reactive immune cells. Their functions include not only regulating and trafficking leukocytes, but also mediating inflammation and pain (García-Velasco and Arici, 1999). The chemokines are classified into two subfamilies, including CXC chemokines (α-chemokines, represented by IL-8) and CC chemokines (β-chemokines, represented by monocyte chemoattractant protein-1 (MCP-1)). Normally, the debris of endometrial cells is expelled in the non-pregnant uterus, triggering menstruation (Kayisli et al., 2002). In primary dysmenorrhea, both the produced MCP-1 and IL-8 can recruit a large number of leukocytes into the endometrium after the withdrawal of progesterone, thereby promoting the shedding of the endometrium (García-Velasco and Arici, 1999). This process attracts inflammatory cells to migrate and infiltrate the site of inflammation (Singh et al., 2021). Elevated plasma chemokine levels have been reported in women with menstrual cycle-associated symptoms compared with healthy women under the regulation of sex hormones (Roomruangwong et al., 2021). In addition, higher serum eotaxin levels have been reported in women with primary dysmenorrhea compared with healthy women (Gul and Celik Kavak, 2018). Thus, dysregulation of chemokines may contribute to the development of primary dysmenorrhea. Furthermore, CD40/CD40L plays important roles in cellular immunity and inflammation, binding of CD40L to its receptor on endometrial cells can lead to overexpression of chemokine IL-8 in endometrial myofibroblasts following progesterone withdrawal (Kelly et al., 2002; Urbich and Dimmeler, 2004). Another study reported that the AMP-activated protein kinase (AMPK) can alleviate dysmenorrhea, by inhibiting the production of chemokines IL-8 and MCP-1 in endometrial stromal cells (Kawano et al., 2021). Collectively, these studies suggest that the excessive secretion of chemokines may serve as potential mediators of pain occurrence in primary dysmenorrhea (Chen et al., 2013).

### 2.9 Neurotransmitters markers: endogenous opioids, 5-HT, endocannabinoids, NMDAR

The endogenous opioid peptides β-endorphin and enkephalin are present in the endometrial secretions of women during reproductive age (Petraglia et al., 1986). Such substances can be produced by immune cells of inflamed tissues and exert analgesic effects (Przewłocki et al., 1992). As an inflammatory disease and a stressful event, primary dysmenorrhea has been associated with dysfunction of opioid peptide-mediated descending pain inhibitory pathways (Heinricher et al., 2009; Bagley and Ingram, 2020). In an animal study, plasma β-endorphin levels were significantly reduced in rats with primary dysmenorrhea compared with normal controls, and this reduction was reversed by herbal-cake-partitioned moxibustion at the Shenque (CV8) acupoint (Chen et al., 2019). Furthermore, a previous review indicated that some interventions such as medicinal plants, drugs and acupressure may exert analgesic effects by upregulating β-endorphin levels (Sharghi et al., 2019). Thus, changes in the concentration of endogenous opioid peptides may be associated with the onset of pain during menstruation.

Serotonergic pathways in the CNS play a crucial role in the modulation of pain transmission and processing (Cortes-Altamirano et al., 2018). Decreased serum serotonergic activity and increased cerebrospinal fluid levels of excitatory amino acids/peptides (e.g., glutamate, substance P) is associated with chronic primary pain (Rekatsina et al., 2020).

As one of the important neurotransmitters, 5-HT can promote analgesia by activating 5-HT receptors (possibly 5-HT2A/2C subtypes) on spinal GABAergic/enkephalinergic interneurons, thereby increasing GABA and enkephalin release (Martins, 2019). An animal study found (Xiaofei, 2016) that the concentration of 5-HT in the hypothalamus of rats with primary dysmenorrhea was lower. Therefore, 5-HT may serve as a potential marker of primary dysmenorrhea as well as one of potential therapeutic targets.

Endocannabinoids have the effect of regulating neurotransmitters and pain, which can reduce nociceptive transmission. The cannabinoid receptor CB1 is associated with pain and is mainly expressed in the dorsal horn of the spinal cord (Finn et al., 2021). 2-Arachidonoylglycerol (2-AG) is the main endocannabinoid, which can activate CB1R located at the presynaptic terminal (Fletcher-Jones et al., 2020). After CB1R is activated, it can regulate voltage-gated channels by inhibiting the influx of Ca^2+^ (Zou and Kumar, 2018) and promoting K^+^ influx to relieve pain. It also inhibits the release of glutamate and nitric oxide, protecting nerves from harmful excitatory stimuli (Zou and Kumar, 2018, review). In addition, cannabinoids can directly act on hyperpolarized activated cyclic nucleotide-gated (HCN1) channels (Mayar et al., 2022). Cannabinoids regulate pain by modulating the expression of substance P and N-methyl-D-aspartate receptor subunit 2B (NR2B) (He et al., 2019). In the study of dysmenorrhea due to endometriosis, it was found that the severity of endometriosis-associated pain was negatively correlated with the level of endogenous cannabinoids in female patients (Sanchez et al., 2016). Elevated levels of 2-arachidonic glycerol (2-AG), a representative of endocannabinoids, mediate pain and are involved in the regulation of dysmenorrhea (Andrieu et al., 2022). Therefore, endogenous cannabinoids may be considered for study as a mediator involved in primary dysmenorrhea.

The N-methyl-D-aspartate receptor (NMDAR) is an important molecule in the dorsal horn of the spinal cord to transmit nociceptive information and enhance the central sensitivity to pain (de Geus et al., 2020). The NMDAR is composed of four subunits, of which the N-methyl-D-aspartate receptor subunit 2B (NR2B) is most closely related to central and peripheral pain (Xu et al., 2020). Pain caused by nerve injury or chronic ischemia activates the presynaptic or postsynaptic NMDAR. At this time, magnesium ions cannot block NMDAR channels, resulting in a large influx of calcium ions, and the expression of NR2B subunits in the spinal cord is upregulated. Moreover, NR2B is phosphorylated, and phosphorylated NR2B promotes calcium influx and pain transmission (Xu et al., 2020; Pan L. et al., 2022; Aiyer et al., 2018). In the study of magnesium and pain, dysmenorrhea has been studied as a clinical disease that magnesium can prevent and treat. It is because that magnesium acts as an antagonist of NMDAR by inhibiting NMDA receptors, which in turn exerts analgesic effects (Shin et al., 2020).

## 3 Conclusion and perspectives

**FIGURE 1:**
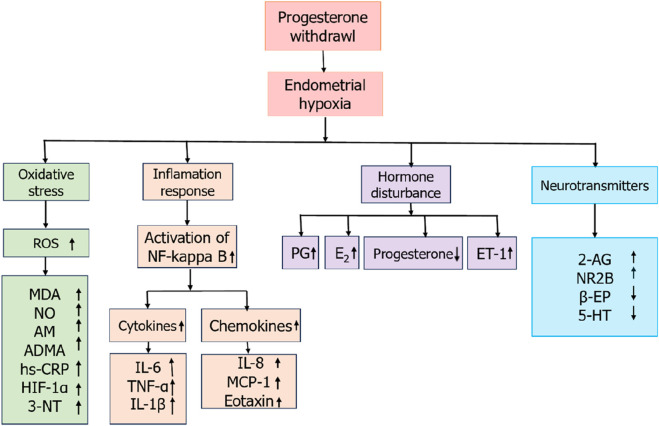
The picture above is a relevant potential marker for primary dysmenorrhea.

Primary dysmenorrhea is a common clinical condition nowadays, but early diagnosis of primary dysmenorrhea is rarely performed in the clinic. The discovery and application of markers can help clinics achieve early diagnosis of primary dysmenorrhea (Figure 1).

Some inflammatory markers of primary dysmenorrhea such as prostaglandins, estradiol and progesterone have been identified. There are still some endothelin-1, cytokines, neuropeptides and oxidative stress markers whose heterogeneity in primary dysmenorrhea has not been thoroughly investigated. More potential biological inflammatory markers of primary dysmenorrhea that have not been identified and measured. These mediators associated with primary dysmenorrhea have not yet been formally incorporated into the clinical diagnosis of primary dysmenorrhea. These mediators still require further clinical research for validation. Therefore, scholars can use metabolomics and proteomics techniques, immunoassay methods, and mass spectrometry coupling techniques in future studies to search for more markers of primary dysmenorrhea. In addition, molecular mediators involved in the inflammatory process of menstruation include lipid mediators, complement and etc. Therefore, lipids and oxylipins may also be considered for study as a marker of lipid mediators associated with primary dysmenorrhea. Additional markers associated with pathological changes in primary dysmenorrhea will be identified by the technical testing of oxylipin. In addition, the search for neurostructural markers and neurofunctional markers of primary dysmenorrhea using brain imaging and neuroimaging techniques is promising. Future research should aim to screen for comprehensive, rational and sensitive markers or combinations of markers, and to seek more precise, specific markers that reflect the occurrence of the corresponding symptoms in dysmenorrhea, in order to identify new therapeutic targets and approaches for primary dysmenorrhea.
